# A provincial cost analysis of electric vehicle operation in China

**DOI:** 10.1016/j.isci.2025.113504

**Published:** 2025-09-07

**Authors:** Bo Li, Mingxia Yang, Gang He, Guangchun Ruan, Jianxiao Wang, Xueqin Cui, Haiwang Zhong, Daniel M. Kammen

**Affiliations:** 1 School of Electrical Engineering, Guangxi University, Nanning 530004, China; 2 Department of Electrical Engineering, Tsinghua University, Beijing 100084, China; 3 Marxe School of Public and International Affairs, Baruch College, City University of New York, New York, NY 10010, USA; 4 CUNY Institute for Demographic Research, City University of New York, New York, NY 10010, USA; 5 Earth and Environmental Sciences, The Graduate Center, City University of New York, New York, NY 10016, USA; 6 Laboratory for Information & Decision Systems, Massachusetts Institute of Technology, Boston, MA 02139, USA; 7 National Engineering Laboratory for Big Data Analysis and Applications, Peking University, Beijing 100871, China; 8 California-China Climate Institute, University of California, Berkeley, Berkeley, CA 94709, USA; 9 Department of Civil and Systems Engineering (CASE), School of Advanced International Studies (SAIS), Johns Hopkins University, Baltimore, MD, USA

**Keywords:** applied sciences, electrical engineering, economics

## Abstract

The transition to all-electric driving is essential for achieving carbon reduction goals in China’s transportation sector, which is significantly influenced by economic costs and emission reduction benefits. In this study, a comprehensive provincial assessment method is developed to quantify the levelized cost of operation (LCOO) for four electric vehicle (EV) types in China, revealing a complex interaction between geographical, economic, and technological factors. We find that the LCOO of EVs ranges from $1.36/100 km for private light-duty vehicles in the northwest region to $26.09/100 km for heavy-duty trucks in the northeast region. The results emphasize the significant disparities in the LCOO of EVs across vehicle types and regions while underscoring the relative importance of various cost components. With the widespread electrification of transportation, there is an urgent requirement for a comprehensive model to disaggregate and estimate the LCOO considering multiple factors, and formulate targeted strategies for expediting EV regional development.

## Highlights

• A provincial assessment method is proposed for evaluating the EV operational costs in China
• Average EV operational costs range from $1.45/100 km for LDVs to $23.59/100 km for HDTs
• Charging options and vehicle features increase the disparity in EV operational cost
• The costs of GHG emissions for EVs increase the heterogeneity of regional EV deployment

## Graphical abstract

**Figure:**
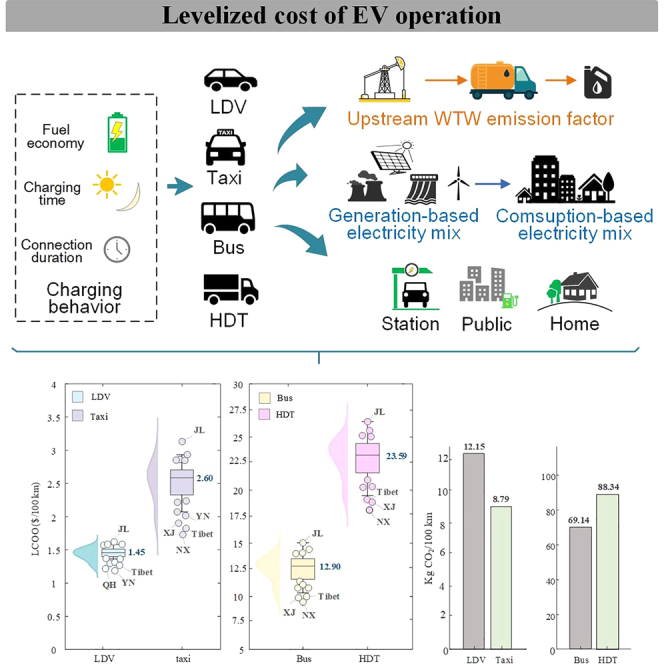

## Introduction

Electric vehicles (EVs) are increasingly emerging as feasible substitutes for traditional internal combustion EVs (ICEVs) due to their lack of tailpipe emissions.^1^^,^^2^ The purchase cost of EVs is relatively high compared to ICEVs at the initial stage, and government subsidies for EVs mitigate purchase cost disparities, enhancing consumer acceptance.^3^ Recently, the sales-weighted average price of EVs before purchase subsidy in China has fallen below that of ICEVs, and approximately 65% of the EVs sold in 2023 were cheaper than their average ICEV equivalent.^4^ With the phase-out of EV purchase subsidies and the price parity between EVs and ICEVs, mass-market consumers focus on operational and maintenance costs to reduce expenses in long-term operations.^5^^,^^6^ Hence, it is necessary to understand the levelized cost of operation (LCOO) of vehicles and the primary influencing factors when making decisions such as the phasing out of government subsidy policies by policymakers and the purchase of EVs by prospective consumers.^7^

Previous studies have acknowledged that fuel costs, such as charging costs, are considered to be the operational cost of EVs.^8^^,^^9^ The LCOO of EVs depends on a variety of factors, such as the retail electricity price, capital costs of EV supply equipment (EVSE), costs of installation and maintenance of EVSE, greenhouse gas (GHG) emissions, and vehicle parameters (e.g., fuel economy and vehicle range).^10^ Each factor is further affected by variables such as the EVSE and vehicle type, charging location, charging schedule, and geographic region of operation. Consequently, the actual operational costs of EVs exhibit disparity between uniform charging cost assumptions due to the extensive heterogeneous variables, resulting in a wide range of potential operating costs for EVs.^9^ With the ambitious decarbonization goal in China, the EV operation costs have not yet been evaluated systematically among various vehicle types and charging options, despite significant heterogeneity in operational costs. Furthermore, the operating costs of EVs in terms of savings compared to those of ICEVs are contributed to by the economy of charging, and the benefits of GHG emissions, which play a pivotal role in facilitating their widespread adoption.^11^

Previous studies have explored the economics of EV charging, which is a significant advantage for the LCOO of EVs in contrast to ICEVs. The charging infrastructure has been shown to have a non-negligible effect on EV sales.^12^ The charging price, as an endogenous factor, should be carefully considered when modeling the charging infrastructure market with direct and indirect factors.^13^ Economic evaluation and business models of charging infrastructures have been studied in China,^14^^,^^15^^,^^16^ the United States,^10^ and Europe.^9^ Furthermore, an economic assessment related to residential, workplace, and rapid charging infrastructures was performed, along with their associated expenditures and revenue.^17^ Additionally, the operational benefits associated with EVs are also derived from the reduction in GHG emissions compared to ICEVs. In China, power generation enterprises purchase carbon emission quotas from the market if they emit more than the allowable amount.^18^ In addition, previous studies have indicated that the implementation of carbon tax policy on vehicles facilitates the synergistic reduction of emissions.^19^^,^^20^ For instance, the GHG emission of road transportation in the European Union achieved an 8%–26% reduction, and one of the effective policies is the combination of carbon taxes with green vehicle incentives to match zero-emission targets.^21^ The implementation of carbon tax policy on transportation has also been examined in other countries, such as India,^22^ the United States,^23^ and China.^24^ The aforementioned cost model, however, ignored the influence of life cycle GHG emissions in quantifying the operational cost of vehicles, leading to a deviation in the operational cost incorporated into the LCOO.

Moreover, various EVs exhibit differences in driving performance and charging characteristics, resulting in variations in their LCOO. The existing studies, however, primarily focused on battery/plug-in light-duty passenger vehicles and did not extend to other vehicle types.^10^^,^^25^ The electrification of heavy-duty trucks is usually regarded as infeasible due to the issues related to long driving distances, high energy use, low specific energy, and high battery costs. However, the economic and technical feasibility of heavy-duty battery-powered electric trucks might have generally been underestimated.^26^^,^^27^ For instance, EVs in California can support 76% of commercial Class 2B-7 vehicle miles traveled.^28^ European Union (EU) studies indicated that electric trucks could cover 71% of ton-kilometers in Switzerland and 38% in Finland.^29^ In addition, the variation in the fuel economy of vehicles based on energy type (e.g., gasoline or electricity), gross vehicle weight (GVW), and the environment is considered to significantly impact the cost of driving an EV.^30^^,^^31^ Many studies have utilized a vehicle’s national average fuel economy^32^ or representative fuel economy vehicles^33^ for their assessments.

To better quantify the economic and environmental benefits of EVs, we develop a database that incorporates numerous parameters related to various vehicle types based on statistical databases^34^ and government regulatory reports.^35^ A comprehensive provincial assessment of the LCOO of four EV types in China is proposed to explore the heterogeneity of charging options, regions, and GHG emissions. The EV types considered include private light-duty vehicles (LDVs), commercial light-duty vehicles (taxis), heavy-duty passenger vehicles (buses), and heavy-duty trucks (HDTs). To better assess the robustness of the LCOO for four EV types, we perform a sensitivity analysis of six input factors related to vehicle kilometers traveled (VKT), electricity price, carbon price, EVSE cost, temperature, and fuel economy. The results highlight the need for a comprehensive model to determine the LCOO distribution and the importance of differentiated strategies for accelerating EV development.

## Results

### Baseline scenario analysis

The base year in this study is set to 2020 due to limitations in data availability. Table 1 shows the key parameters in the baseline scenario, which are used to quantify the LCOO for four types of EVs and ICEVs in China. The key factors, including vehicle parameters, EVSE parameters, and regionally heterogeneous parameters, are explored in the baseline scenario.

**Table 1:** The parameters in the baseline scenario

Factor	LDV	Bus	HDT	Taxi
FE (BEV, kWh/km)	0.134	1.14	1.46	0.19
FE (gasoline, L/100 km)	6.82	37.2	49.2	6.0
Annual VKT (km)	12,000	120,000	40,000	120,000
Lifetime (years)	10	10	10	10
GVW (tons)	1.48 ICEV, 1.51 EV	18	26	1.2 ICEV, 2.4 EV
Battery capacity (kWh)	26–100 (50)	170–340 (310)	210–500 (370)	47–80 (50)
EVSE: EV ratio	1:1.25 residential, 1:7 public	1:1.6 public	1:1 public	1:4 public
Charging mix	81% residential, 9% public, 10% DCFC	50% 32 kW, 50% 320 kW	50% 40 kW, 50% 400 kW	30% 60 kW, 70% 120 kW
Electricity price	residential and commercial	commercial	commercial	commercial
EVSE costs ($/pile)	encompassing equipment costs, installation costs, and O&M costs			
Fuel price	gasoline prices range from $1.01/L in Xinjiang to $1.07/L in Sichuan.			
Carbon price	the national average price for carbon emissions is $7/ton.^36^			
Emission factor	the WTT and TTW GHG emission factors of gasoline are 0.9514 and 2.2869, respectively. The upstream GHG emission factor and combustion emission factor of coal are 0.1679 and 2.772, respectively.^37^			
Discount rate	the discount rate is assumed to be 7%.			
Battery degradation loss	the annual battery degradation rate is assumed to be 0.71%.^38^			

#### Vehicle parameters

A fair comparison of different vehicle types necessitates considering those of similar size and identical performance. This paper determines the GVW ranges for LDVs, taxis, buses, and HDTs by referring to the vehicle classifications of the EU and Federal Highway Administration, and then, the average GVWs of these vehicle types are calculated. In addition, a Chinese database of fuel economy for various vehicle types based on statistical databases^34^ and government regulation reports^35^ is developed to estimate the fuel economy under a specific average GVW of vehicles (Table S1). Then, based on fuel economy and GVW, we determined the specific models of LDVs, taxis, buses, and HDTs for EVs and ICEVs (Figure S3). Besides, we assume that the VKT and lifespan of EVs and ICEVs are identical for a direct comparison.^39^

#### EVSE parameters

There are three EVSE types based on the charging power, namely, L1 (home charger, <7 kW), L2 (7–30 kW), and L3 (>60 kW), as shown in Table S2. The EVSE costs, including equipment, installation, land, and operation and maintenance (O&M) costs, are significantly influenced by the charging power (Table S3; Figure S4). According to the “Guidelines for the Development of Electric Vehicles Charging Infrastructure” reported by the NDRC,^40^ the planning targets of public EVSE: EV ratios by vehicle type are 1:1.6 for buses; 1:1 for taxis; 1:1 for trucks; and 1:1 for private, dedicated, and public institutions (Table S4). The charging mix represents the share of charging power at each charging location based on the data reported by the Electric Power Research Institute^41^ (Tables S5 and S6).

#### Regionally heterogeneous parameters

Private LDVs are recharged at home and in public places, with corresponding residential and commercial electricity prices. Other vehicle types only recharge in public areas with commercial electricity prices. Time-of-use prices are implemented to encourage residential/commercial consumers to utilize electricity at night or during off-peak periods. Detailed information regarding prices is provided in Figures S5 and S6. The national average carbon price is utilized as the input parameter to avoid significant regional fluctuations.^36^

Scenarios with various charging powers in the charging mix, as shown in Table 2, are explored to estimate potential changes in the LCOO of EVs. The charging power at different charging locations (home, workplace, and public place) varies, which significantly affects the LCOO of EVs. In the low-charging-power (LCP) scenario, EV users prefer to recharge their vehicles at a low charging power because of lower charging costs. Instead, the high-charging-power (HCP) scenario is deemed the least favorable option for EV users who lack adequate charging infrastructure and time for charging. The assumptions of the medium-to-low-charging-power and medium-to-high-charging-power scenarios are between those of the LCP and HCP scenarios to represent more likely charging options. The baseline scenario in Table 1 is derived from real-world situations and is chosen based on actual range data, reflecting the existing technology.

**Table 2:** The key input assumptions for five charging scenarios

Charging mix	LDV	Bus	HDT	Taxi
LCP	100% residential 0% public, 0% DCFC	100% 32 kW 0% 320 kW	100% 40 kW 0% 400 kW	100% 60 kW 0% 120 kW
Medium-to-low-charging power	90% residential 4% public, 6% DCFC	75% 32 kW 25% 320 kW	75% 40 kW 25% 400 kW	75% 60 kW 25% 120 kW
Baseline	81% residential, 9% public, 10% DCFC	50% 32 kW 50% 320 kW	50% 40 kW 50% 400 kW	30% 60 kW 70% 120 kW
Medium-to-high-charging power	37% residential 50% public, 13% DCFC	25% 32 kW 75% 320 kW	25% 40 kW 75% 400 kW	15% 60 kW 85% 120 kW
HCP	5% residential 65% public, 30% DCFC	0% 32 kW 100% 320 kW	0% 40 kW 100% 400 kW	0% 60 kW 100% 120 kW

### Charging options and vehicle technical characteristics increase the LCOO disparity among EVs

**Figure 1:**
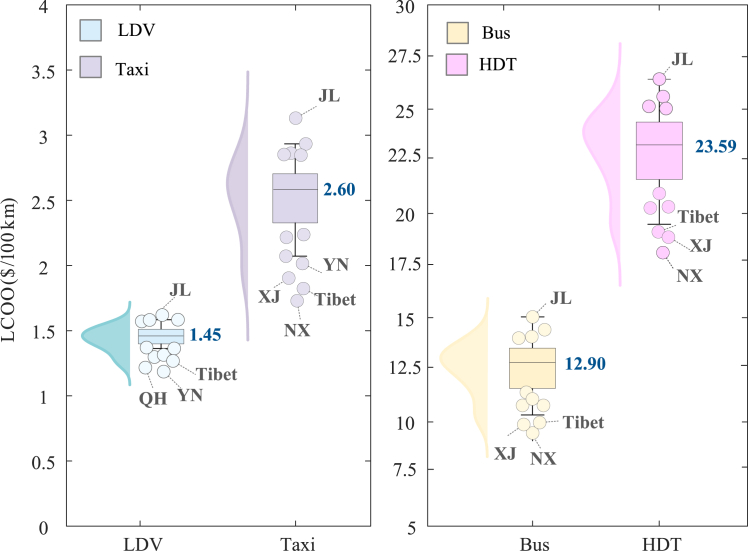
The provincial distribution of the LCOO for the four EV types in the baseline scenario The boxplots show the distribution of the LCOO for the four EV types across provinces in China. The kernel density curves are utilized to illustrate the concentrated distribution pattern of LCOO, indicating that a high degree of curve fluctuation corresponds to a high percentage of provincial LCOO. The values denote the national average LCOO for four EV types. Moreover, the provinces that exhibit significant deviations from the average LCOO are denoted with data points. QH, Qinghai; SD, Shandong.

Figures 1 and S1 show the provincial distribution of the LCOO for four EV and ICEV types in the baseline scenario. The results show that the provincial LCOO of EVs is substantially lower than that of ICEVs across vehicle types. Specifically, the average LCOOs of EVs are $1.45/100 km for LDVs, $2.60/100 km for taxis, $12.90/100 km for buses, and $23.59/100 km for HDTs, while those of ICEVs are $7.30/100 km, $6.42/100 km, $39.82/100 km, and $52.67/100 km, accordingly. These significant disparities mainly stem from the higher fuel prices and GHG emission intensity.

In addition, provinces such as Ningxia (NX), Shandong, Tibet, Xinjiang (XJ), Yunnan (YN), and Jilin (JL) demonstrate comparable deviations in LCOO across EV types. For LDVs, private passenger vehicles in YN, Qinghai, and Tibet provinces display the lowest LCOOs ranging from $1.21/100 km to $1.25/100 km because of the low residential electricity prices with an average of $0.062/kWh and relatively low GHG emission intensity of the power grid (0.84–1.73 kg/100 km) in these areas. NX and XJ provinces have comparatively lower LCOOs for taxis ($1.83/100 km and $2.00/100 km), buses ($9.22/100 km and $9.74/100 km), and HDTs ($18.02/100 km and $19.01/100 km) among the three commercial vehicles. This can be attributed to the lower commercial electricity price ($0.077/kWh and $0.064/kWh) resulting from abundant resources. JL province has the highest LCOO, ranging from $1.61/100 km for electric LDVs to $27.43/100 km for electric HDTs as a result of its notably high electricity prices and the impact of cold weather on fuel consumption.

**Figure 2:**
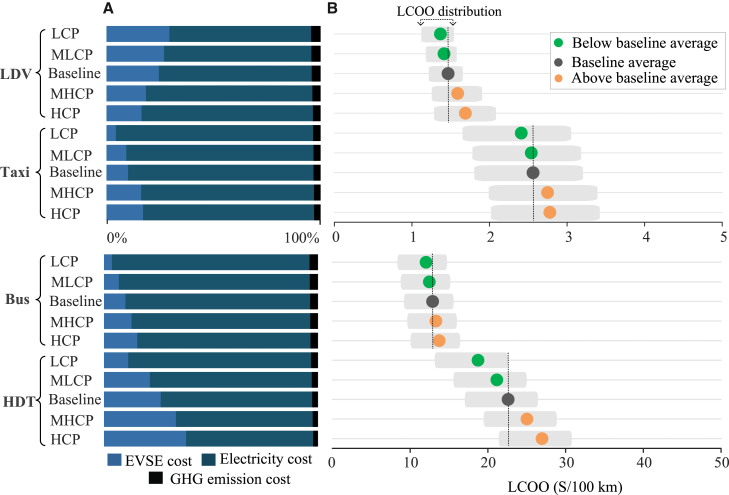
The cost components and distributions of the LCOO of EVs in five charging scenarios (A) The cost components of LCOO for the four EV types in five charging scenarios, encompassing EVSE costs, fuel costs, and GHG emission costs. (B) The distributions of the LCOO across five charging scenarios for the four EV types. The average LCOO of EVs for each hypothetical scenario is highlighted with dots. The gray dots and vertical dotted lines represent the average LCOO of EVs in the baseline scenario. The average LCOO of EVs for charging scenarios below the baseline are denoted as green dots, while scenarios with LCOO values higher than the baseline are denoted as orange dots. Both (A) and (B) share the same *y* axis representing the charging scenarios.

In Figure 2A, we compare the cost components of LCOO to explore the differences in drivers for four EV types and charging scenarios. The electricity cost dominates the LCOO, accounting for a substantial proportion ranging from 56.31% to 92.31%. Conversely, the GHG emission cost has the lowest proportion of LCOO, ranging from 2.18% to 4.49%. There are also significant disparities in the proportion of cost components among the various EV types. HDTs exhibit the highest EVSE cost proportion, varying between 11.30% and 41.51%, while for buses, this proportion is the lowest, ranging from 3.68% to 15.55%. This disparity suggests that HDT charging strategies have huge potential to be optimized to reduce the LCOO. For LDV, EVSE cost is more sensitive to charging options in LCP scenarios. For instance, when home charging increases by 19%, the proportion of EVSE costs increases from 26.66% to 29.36%. In addition, advancements in EVSE technologies coupled with the transition to a more sustainable and low-carbon power grid would decrease EVSE and GHG emissions costs.

Second, Figure 2B highlights that the difference in the LCOO distribution highly depends on the charging mix. The LCP scenario is the most economical option, with the LCOO ranging from $1.37/100 km to $18.75/100 km. In contrast, in the HCP scenario, the LCOO of EVs ranges from $1.73/100 km for LDVs to $28.43/100 km for HDTs, which is 10.16%–34.31% higher than that in the baseline scenario. The results can be attributed to the high utilization of lower charging power in the LCP scenario, which is associated with the lower capital costs of EVSE. Note that the LCP scenario is subject to significant limitations, such as the long charging duration of EVs. For instance, in the LCP scenario, the charging duration for four EV types with a 60 kWh capacity increases 2.38 h for LDVs, 16.8 min for taxis, 40.5 min for buses, and 32.4 min for HDTs when the state of charge is increased from 20% to 100%, in comparison to the baseline scenario. In public places, short charging durations are required for EV users to reduce mileage anxiety. Therefore, it is essential to select a suitable charging power to effectively balance the LCOO and charging duration requirements.

There are differences in the LCOO of vehicle EV types within the same charging scenario (Figure 2B). For instance, in the HCP scenario, the LCOO of HDTs is $28.43/100 km, which is significantly greater (19.28%) than that in the baseline scenario. However, in the same scenario, the LCOO of buses is $13.73/100 km, which is moderately higher (20.52%) than that in the baseline scenario. The significant difference in LCOO among various vehicle types can be attributed to the greater charging power compared to that in the baseline scenario. Moreover, the variations in electricity prices for the four vehicle types dominate the LCOO changes. The national average price of commercial electricity is $0.1521/kWh, which is 65.78% higher than the residential electricity price during peak hours. Home chargers offer a considerable benefit to electric LDVs by eliminating the dependence on public charging infrastructure and enabling them to capitalize on low residential electricity prices. Thus, LDVs have the lowest electricity cost for LCOO (69.15%) in the baseline scenario. Due to the requirement for fast charging speeds and security, taxis typically prefer to select basic or peak commercial electricity prices, resulting in a higher electricity cost proportion for the LCOO (85.53%) compared to that for LDVs in similar scenarios (Figure 2A).

### The GHG emission cost of EVs increases the heterogeneity of regional EV deployment

**Figure 3:**
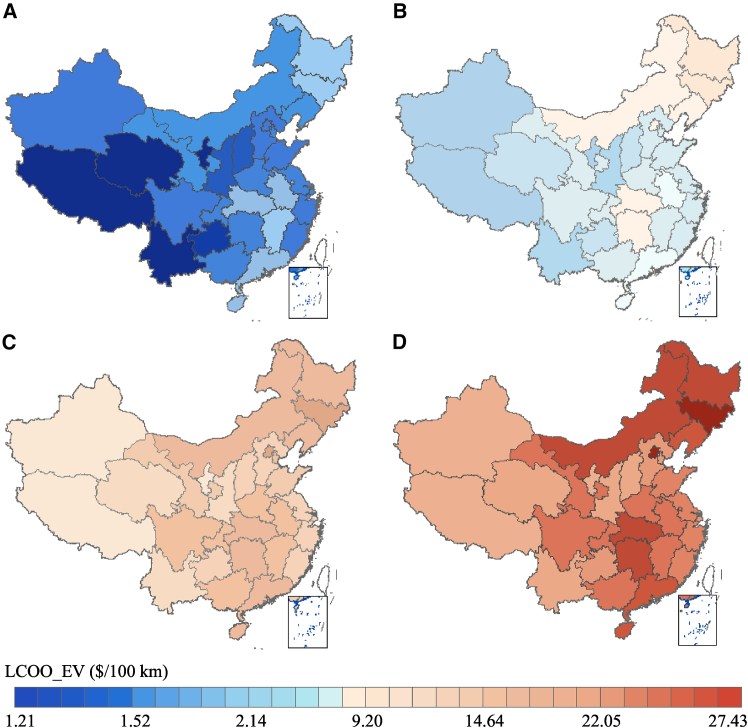
The provincial variation in the LCOO for the four EV types in the baseline scenario (A) Private LDVs. (B) Taxis. (C) Buses. (D) HDTs. The low LCOOs of EVs are predominantly clustered in the western area. The Northeast region has a comparatively higher LCOO for EVs due to the colder climate and greater coal-based power generation than in other regions. The eastern area, characterized by general economic prosperity, is also associated with a high LCOO for EVs.

The LCOOs of four EV and ICEV types in each province under the baseline scenario are shown in Figures 3 and S2, respectively. The distribution of the LCOO of EVs displays significant regional heterogeneity compared to that of ICEVs. The northeast region generally has the highest LCOO for EVs, varying from $1.55/100 km for LDVs to $26.09/100 km for HDTs. In contrast, the LCOO of EVs in the northwest region ranges from $1.36/100 km for LDVs to $20.35/100 km for HDTs, which is the lowest level in the country and is 12.31%–28.85% lower than that in the northeast region. For ICEVs, the northeast region exhibits the lowest LCOO, with a range of $7.26/100 km for LDVs to $52.37/100 km for HDTs, while the southern region displays the highest LCOO, varying from $7.39/100 km for LDVs to $53.32/100 km. This indicates that low regional heterogeneity exists in the LCOO of ICEVs, resulting from the slight provincial variations in gasoline prices. In addition, heterogeneity in the LCOO of EVs exists not only among different regions but also among provinces within the same regions. For instance, in the southern region, Yunnan has the lowest LCOO of LDVs at $1.21/100 km, yet the LCOO of LDVs in Guangdong is relatively high nationally at $1.57/100 km, which can be attributed to the higher electricity prices. Furthermore, the significant disparities in the provincial LCOOs of the four EV types stem from the regional heterogeneity of electricity prices and the GHG emission intensity.

The LCOO varies significantly by province, which can be attributed to the regional heterogeneity of electricity prices depending on the heterogeneous electricity supply mix. Figure S5 shows the provincial time use of electricity prices. In China, due to spatially uneven, imbalanced economic development and the distribution of the electricity supply and demand, the eastern area needs to purchase electricity from the western area to meet the electricity demand, resulting in higher electricity prices. For instance, in the southern region, Guangdong province has a high commercial basic electricity price of $0.1038/kWh because the local area power supply is insufficient, and it purchases large amounts of electricity from Yunnan, Guizhou, and other provinces. High electricity prices result in a more expensive average LCOO in Guangdong, such as $1.57/100 km for electric LDVs. There are several similar provinces with high electricity prices, such as Beijing, Hubei, and Hunan provinces.

**Figure 4:**
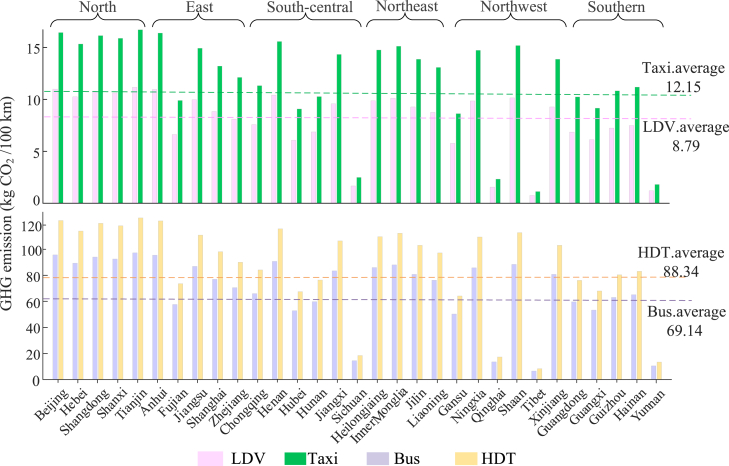
The provincial GHG emission intensity for four EV types The dashed lines represent the average GHG emission intensity at the national level for four EV types. The columns represent the GHG emission intensity of EVs across different provinces, with the purple columns (LDVs), yellow columns (taxis), pink columns (buses), and green columns (HDTs) corresponding to different vehicle types.

Figures 4 and S1B show a comparison of the average GHG emission intensity among the four EV and ICEV types. The GHG emission intensity of EVs is substantially lower than that of ICEVs for the same vehicle types. The GHG emission intensity of EVs ranges from 8.79 kg CO_2_/100 km for electric LDVs to 88.34 kg CO_2_/100 km for electric HDTs. However, the GHG emission intensity of ICEVs ranges from 22.46 kg CO_2_/100 km for gasoline LDVs to 162.72 kg CO_2_/100 km for gasoline HDTs. Compared to gasoline LDVs, the LCOO of electric LDVs is the most significant in terms of the proportional reduction in the GHG emission cost, at 60.87%. In contrast, the shift from gasoline taxis to electric taxis results in the lowest proportional reduction in GHG emission costs at 35.79%. These findings suggest that deploying electric LDVs would have considerable GHG emission benefits, and similar shifts to the other three commercial vehicles would result in lower GHG emission benefits than those for LDVs. Additionally, this implies that policymakers could introduce carbon taxes to spur the large-scale development of EVs in different regions due to the GHG emission disparity between EVs and GVs.

Figure 4 also displays the provincial GHG emission intensity for the four types of EVs. Moreover, deep grid decarbonization significantly reduces GHG emission costs. For instance, 95.37% of the electricity generated comes from clean power in Tibet, while in Tianjin, this proportion is only 3.68%. As a result, Tianjin has the highest GHG emission cost at $ 0.44/100 km, which is 14.67 times greater than that in Tibet ($ 0.03/100 km). The trends in Yunnan, Sichuan, and Qinghai provinces are similar to those in Tibet.

**Figure 5:**
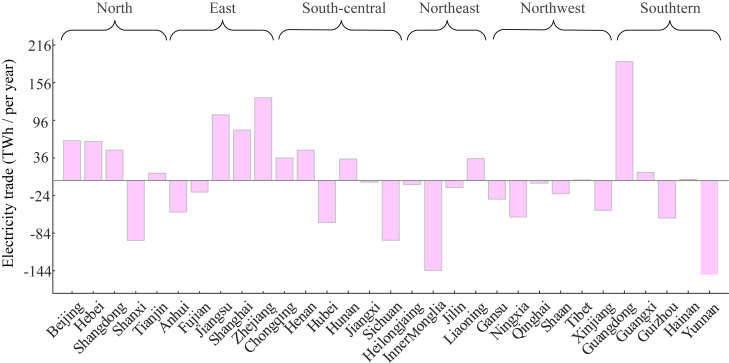
Annual provincial electricity trade volume The positive and negative values represent the electricity imported and exported by the provinces, respectively. The provinces that import a significant amount of electricity are mainly coastal areas, such as Guangdong, Zhejiang, and Jiangsu provinces. The provinces that export a large amount of electricity are mainly located in the western areas, such as Yunnan, Sichuan, and Inner Mongolia.

In addition, exporting a large amount of clean electricity from other provinces would reduce GHG emission costs. The amount of electricity traded for each province is optimized by minimizing the national electricity cost and the equilibrium of the total power generation across all provinces, as shown in Figure 5. Thus, provinces with electricity shortages have prioritized purchasing electricity from neighboring provinces. The proportion of clean electricity in Shanghai increased by 14.83% because of the large amount of highly renewable electricity transmitted to Shanghai from Hubei and Sichuan provinces. This results in a 16.85% reduction in GHG emission costs. Conversely, Beijing imports a large amount of coal-generated electricity from Inner Mongolia and Hebei provinces, resulting in a considerable GHG emission cost of $0.43/100 km. Therefore, developing renewable energy has considerable effects on GHG emission reductions for EVs, especially in regions with deep grid decarbonization. Furthermore, LDVs are associated with the highest proportion of GHG emission costs at 4.23%, while the lowest value is observed for HDTs, at 2.62% in the baseline scenario (Figure 2A). Deploying electric LDVs in economically prosperous regions would significantly reduce the GHG emission cost resulting from accelerating the decarbonization grid transition. Deploying electric HDTs would result in a considerable reduction in the GHG emission cost in the western region because of deep grid decarbonization.

### Sensitivity analysis

**Figure 6:**
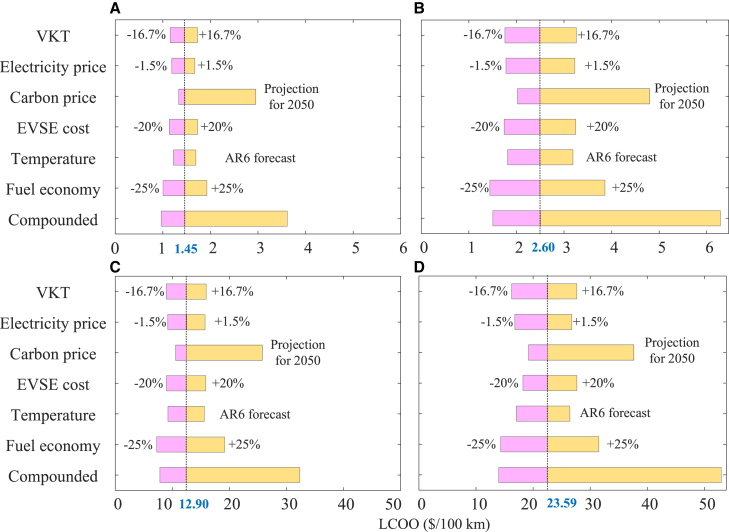
Sensitivity of the LCOO for four EV types (A) Private electric LDVs. (B) Electric taxis. (C) Electric buses. (D) Electric HDTs. The dotted lines represent the average LCOO of EVs in the baseline scenario. The “Compounded” bar at the bottom represents the LCOO range when the factors of VKT, electricity price, carbon price, EVSE cost, temperature and fuel economy vary simultaneously.

To better assess the robustness of the LCOO of EVs, a sensitivity analysis is performed on six input factors related to the VKT, electricity prices, carbon prices, EVSE costs, temperature, and fuel economy. Based on the characteristics of input parameters, the sensitivity analysis ranges are determined. Figure 6 summarizes the sensitivity of the LCOO for four types of EVs.

The LCOO is most sensitive to differences in carbon prices, though the proportion of GHG emission costs in the LCOO is not significant in the baseline scenario. The carbon trading market of China has just been established. The operation of the market is still being constantly adjusted according to actual conditions, leading to significant fluctuations in carbon prices.^36^ The carbon price starts to increase rapidly and then rises only moderately after emission neutrality has been reached to keep the temperature increase well below 2°C without large-scale deployment of carbon dioxide removal.^42^ Projected 2050 average carbon prices in China range reaching $117/ton,^43^ leading to an average increase in the LCOO of 68.3% for electric LDVs, 60.00% for buses, 41.92% for HDTs, and 51.92% for taxis. Despite the significant expansion of renewable power generation currently, China still depends substantially on coal power generation.^44^ This dependence on coal power generation, coupled with government regulation, plays an important role in maintaining stable electricity prices in China. Therefore, the sensitivity of LCOO to electricity price is minimal. When the electricity price changes by ±1.5%,^45^ the LCOO of the electric LDVs, buses, HDTs, and taxis ranges from −1.07% to +1.01%, −1.35% to +1.24%, −1.06% to +0.97%, and −1.72% to +0.84%, respectively.

The LCOO is also sensitive to fuel economy, as illustrated by the isolated impacts on a ±25% change in Figure 6,^46^ which means that an improvement in fuel economy of EVs can significantly lower the LCOO. In the future, advancements in battery performance are expected to result in progressive improvements in fuel economy. Furthermore, the government has strict fuel economy requirements for EVs, established in various plans and standards, such as the Corporate Average Fuel Consumption and New Energy Vehicle (CAFC&NEV) Credit Regulation.^47^ Therefore, consumers should have a positive attitude toward the impact of future fuel economy on the LCOO of EVs. LCOO exhibits relatively low sensitivity to temperature compared to the baseline scenario. However, according to the Sixth Assessment Report of the Intergovernmental Panel on Climate Change, the global mean surface air temperature would increase 1.5°C or even beyond within the next 20 years as global climate change intensifies.^48^ The impact of temperature on the LCOO warrants attention.

The “Compounded” bar in Figure 6 shows the variations in the LCOO for the four EV types under the effects of multiple factors. In the worst scenario, the LCOO for the four EV types increases significantly by 115.56% for LDVs, 103.39% for buses, 84.20% for HDTs, and 93.01% for taxis. The average LCOO of LDVs is significantly influenced by the interaction of multiple factors, whereas the impact on HDTs is relatively slight. Conversely, the LCOO for the four EV types declined slightly in the cheaper scenario, as the lower impact of other factors is partially offset by higher future carbon prices. The sensitivity analysis of primary factors signifies that the LCOO of EVs is already more cost-attractive than the operational cost of comparable ICEVs, even in the worst scenario. The combined input of multiple factors results in a significant coupling effect on the LCOO, which highlights the need for a comprehensive model to determine the variation in the LCOO considering multiple factors.

### Conclusion

**Figure 7:**
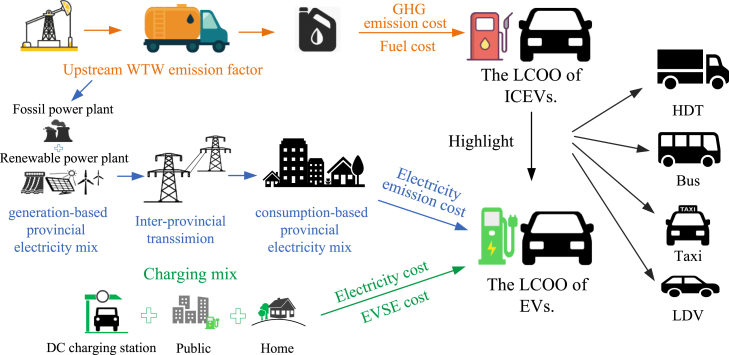
Regional quantitative model of the LCOO for four vehicle types The LCOO of ICEVs consists of fuel costs and GHG emission costs, which reflect the fuel consumption and emissions generated throughout the vehicle’s life cycle per kilometer traveled. The LCOO of EVs includes the GHG emission costs, electricity consumption costs, and EVSE costs. Considering interprovincial electricity trade, the GHG emission intensity of electricity is determined based on a consumption-oriented electricity mix. The charging locations include home, public, and DC fast charging stations.

This study fills a significant research gap in the areas of EV cost analyses based on a comprehensive assessment of the levelized operational cost for four types of EVs in China, revealing a complex interaction between geographical, economic, and technology factors, as shown in Figure 7. Additionally, an in-depth sensitivity analysis is conducted to assess the impact of varying factors on the LCOO of four EV types. The result emphasizes the significant disparities in the LCOO of EVs across different regions and vehicle types while also underscoring the relative importance of various cost components. With the widespread electrification of transportation, the LCOO of EVs should be disaggregated and estimated from a perspective that considers multiple factors to provide a comprehensive quantification.

LDVs, electric private passenger vehicles, have the most economical LCOO compared to other commercial vehicles ($1.45/100 km), resulting from the lowest charging power and electricity prices. Conversely, electric HDTs have the most expensive LCOO of $23.59/100 km, primarily due to their fast charging requirements and high fuel consumption. The disparities in the LCOO largely stem from variations in performance (e.g., fuel economy) and charging mix, which in turn affect the adoption of different EV types. In the baseline scenario, the LCOO is composed of EVSE (11.19%–29.51%), electricity (67.87%–86.26%), and GHG emission cost (2.62%–4.23%). Furthermore, variations in charging power and electricity price across different charging scenarios lead to discrepancies in the LCOO of EVs, and this variability provides EV users with a reference for operational costs.

Moreover, the GHG emission reduction benefits of four EV types are examined to formulate province-specific development strategies according to the regional decarbonization level. The development strategies design of regional schemes needs to effectively articulate the heterogeneity of provinces in terms of GHG emission reduction. The electrification of LDVs offers the most effective GHG emission reduction effect. The environmental benefits of electric buses and HDT cannot be overlooked due to their significant emission per kilometer. In addition, the LCOO can be reduced in regions with abundant renewable electricity, stemming from lower electricity prices. However, electricity prices are high in cold-weather regions that rely on coal-fired power generation, such as the north and northeast regions, and thus, the potential LCOO reduction is modest. To achieve the GHG emission reduction goals of the transportation sector through EV development, the north and northeast regions should prioritize decarbonizing the power grid. Furthermore, deploying electric LDVs in economically prosperous regions would significantly reduce the GHG emission cost resulting from accelerating the decarbonization grid transition. Deploying electric HDTs would result in a considerable reduction in the GHG emission cost in the western region because of deep grid decarbonization.

## Discussion

Technological advancements, favorable economic cost and policies, and increasing awareness of the environment have driven the transition from ICEVs to EVs, paving the way for the achievement of a net-zero GHG emission future for the transportation sector.^49^ China has implemented multiple policies to promote EVs, such as subsidies for EV purchases,^3^ the CAFC&NEV Credit Regulation,^47^ and the acceleration of charging infrastructure.^50^ To effectively facilitate the widespread adoption of EVs for mitigating GHG emissions, a better understanding of the LCOO of vehicles and the primary influencing factors is crucial.

For users who are able to install home chargers, the residential charging option corresponding to the LCP scenario is particularly appealing. Conversely, the EV users residing in urban apartments may lack access to residential charging options and may depend predominantly on public charging infrastructure. Additionally, research shows that urban groups tend to have lower rates of home charging.^51^ Although China has implemented multiple policies to accelerate the development of charging infrastructure, such as subsidies for infrastructure projects,^50^ the current progress of infrastructure has not aligned with the increase in EVs. Consequently, this disparity makes it difficult for some groups to access public EV infrastructure, impeding mass adoption. In addition, the gap in these policies primarily emphasizes broader implementation, neglecting specific characteristics across various regions. The LCOO of vehicles in our analysis identifies significant regional heterogeneity and the urgent requirements to enhance accessibility, highlighting the necessity for local governments to formulate tailored public infrastructure deployment. An effective measure is the strategic development of public charging infrastructure with different charging characteristics in economically disadvantaged areas to support EV deployment strategies, ultimately ensuring charging accessibility for users of various vehicle types.

For prospective EV users, considerable heterogeneity in operational costs effectively reflects the intrinsic economic and environmental benefits of EVs versus ICEVs. With the reduction in the purchase cost of EVs, the operational costs have emerged as a significant concern for prospective purchasers of EVs. However, the existing heterogeneity of the LCOO may not strictly be transmitted to the end-consumers, as the operators for electricity, fuel, and charging infrastructure may set different prices for various regions, particularly in the short term or within an imperfect market. These pricing schemes may be contingent upon the historical evolution of charging infrastructure, price regulations implemented at the national level, or even the stability of the markets. Additionally, for the publicly available commercial infrastructure operated by the same operator, the charging prices or fuel prices may actually remain relatively uniform across various regions, despite disparities in utilization, to maintain competitiveness.^9^

The carbon emissions trading is a powerful tool for facilitating the transition of the electricity generation sector toward a cleaner and more innovative power grid, and this transition will directly influence the future GHG emission trends of EVs. When enterprises bid for quotas determined by marginal abatement costs, carbon prices indicate their scarcity. Conversely, the scale of emission reductions achieved by various schemes can be effectively described by the carbon price levels. The implementation of carbon prices can convey reduction signals to emitters and urge them to take action, highlighting the effectiveness of carbon pricing schemes in reducing GHG emissions including those associated with both EVs and ICEVs. Evidence for the Guangdong province indicates that the signal has made significant contributions to the achieved emissions reductions in the context of the introduction of the ETS pilots in China.^52^ Besides, some studies indicate that an increase in the carbon prices leads to larger emissions reductions than an increase in the fuel price of the same size.^52^ As the international community intensifies efforts to address climate change, higher carbon prices have been proven to be justifiable by the presence of nonnegligible impacts below the climate target or the consideration of risk and uncertainty.^42^ Therefore, in order to fully achieve the carbon reduction goals of the transportation sector, it is imperative to more effectively integrate carbon pricing with other policies. Specifically, we should explore the potential synergies among various policies, with a particular emphasis on the interplay between carbon pricing, the transformation of renewable energy grids, and emissions regulation in specific industries.

Over recent decades, driven by the need for self-sufficiency, GHG emission reduction, and the increasing geopolitical risks, China’s energy landscape is undergoing a transformation, gradually transitioning toward a clean power grid characterized by a high proportion of renewable energy. China has deployed a high proportion of renewable energy generation capacity, particularly in photovoltaic and wind power, with even more ambitious growth prospects in the future. However, the gradual phase-out of coal-fired units and the construction of renewable energy under decarbonization goals can lead to a reduction in the wholesale market price, exacerbating the volatility of electricity prices.^53^ This phenomenon has become more pronounced, especially as PV and wind deployments continue to expand. While this may be advantageous from the consumer’s perspective, it may adversely affect other producers responsible for reserve capacity and balancing services to the power system.^53^ Consequently, it becomes crucial to undertake a careful analysis of the electricity price impacts and to formulate targeted policies. For instance, in the existing power grid management framework of China, it is recommended to optimize the capacity and ancillary services markets to ensure reasonable investment returns, ultimately effectively balancing the stability, economy, and low-carbon goals of power system.^54^ Besides, evidence shows that the phase-out of ICEVs is negatively associated with gasoline prices, due to higher gasoline prices increasing the costs of ICEVs, leading to a potential decrease in consumer preference for this type of vehicle.^55^

This study not only offers specific LCOO values of four EV types for each province in China but also provides general insights into the broader implications for other countries that are developing EVs. First, although the specific values may vary for different countries, such as electricity prices and gasoline prices, the methodology presented can be utilized to calculate the LCOO in regions beyond China. Given the differences in underlying assumptions across countries, the reported LCOO values will undoubtedly vary. The provincial assessment of the LCOO of vehicles is based on a range of scenarios that may be similar to those in other countries. In addition, the sensitivity analysis of the LCOO for four types of EVs reveals an extensive range of uncertainties in the parameter values, and the results highlight the universality of the methodology. Second, strategies for advancing the growth of EVs by considering the LCOO for various EV types in conjunction with regional attributes are outlined, offering insights for other countries and regions.

### Limitations of the study

There are some limitations that can be ameliorated in future research. Each LCOO-related factor, such as the electricity price and the capital and installation costs of EVSE, changes over time. When accounting for the sensitivity of the LCOO to different factors, shifts in these factors could be forecasted according to the actual situation in response to the increased electrification of the transportation sector. In addition, the technical characteristics of some typical vehicles are used in the baseline scenario to quantify the provincial LCOO of vehicles, which results in a certain deviation compared to the results obtained using the characteristics of various specific vehicles in real-world situations. This study is based on monthly electricity generation and consumption, and thus, the evaluated GHG emission intensities of provinces only reflect the monthly average levels. When more detailed data become publicly available, further analysis at daily and hourly levels could be conducted to improve accuracy.

## Resource availability

### Lead contact

Further information and requests should be directed to and will be fulfilled by the lead contact, Daniel M. Kammen (kammen@jhu.edu).

### Materials availability

This study did not generate new unique materials.

### Data and code availability

• All underlying data used in this paper are available in the main text or the supplemental information or their sources have been clearly stated.
• This paper does not report original code.
• Any additional information required to reanalyze the data reported in this paper is available from the lead contact upon request.

## Acknowledgments

This work was supported in part by the 10.13039/501100018571Specific Research Project of Guangxi for Research Bases and Talents (GKAD23026216), the Guangxi Natural Science Foundation (2025GXNSFBA069263), the 10.13039/501100001809National Natural Science Foundation of China (52122706 and 72104142), and the Major Project of Talents of Guangxi Zhuang Autonomous Region.

## Author contributions

Conceptualization and methodology, B.L.; visualization and writing, M.Y.; formal analysis, G.R. and G.H.; formal analysis and writing, J.W.; investigation, X.C.; funding acquisition, B.L.; supervision, H.Z. and D.M.K. All authors have read and agreed to the published version of the manuscript.

## Declaration of interests

The authors declare no competing interests.

## STAR★Methods

### Key resources table

**Table:** 

REAGENT or RESOURCE	SOURCE	IDENTIFIER
**Deposited data**		

China’s provincial gasoline prices	National Development and Reform Commission	https://www.ndrc.gov.cn/xwdt/ztzl/gncpyjg/202012/t20201217_1293038.html
China’s provincial electricity price	National Development and Reform Commission	https://www.ndrc.gov.cn/
vehicle information	China Automobile Fuel Consumption Inquiry System	https://yhgscx.miit.gov.cn/fuel-consumption-web/mainPage
China's provincial electricity generation, consumption	National Bureau of Statistics	https://data.stats.gov.cn/easyquery.htm?cn=A01
Equipment cost information for EVSE and type	JD online shopping website	https://www.jd.com/
China’s provincial temperature	China Weather Network	https://www.weather.com.cn/

**Software and algorithms**		

MATLAB	MathWorks	https://www.mathworks.com/products/matlab.html

### Experimental model and study participant details

This study proposes a provincial assessment method to quantify the levelized operational cost for four EV types in China. All vehicle data and province data are sourced from publicly published materials, as shown in Table 1 and the key resources table.

### Method details

#### Levelized cost overview of EV operation

In this study, a regional quantitative model for assessing the LCOO across four vehicle types is proposed, as shown in Figure 7. This study focuses on the LCOO of vehicles, encompassing the capital and operating costs related to EVSE (only EV), fuel costs (e.g., electricity and gasoline), and GHG emission costs. The specific vehicle types considered are (1) private light-duty passenger vehicles (LDVs), (2) heavy-duty passenger vehicles (buses), (3) heavy-duty trucks (HDTs), and (4) commercial light-duty passenger vehicles (taxis). All types of vehicles offer both traditional gasoline-powered and electric-powered modes. A sensitivity analysis was conducted to explore the factors that influence the LCOO and identify the primary influences on the electricity price, fuel economy, EVSE cost, and temperature.

#### Levelized cost of operation (LCOO)

The LCOO represents the levelized cost of the vehicle operation, including the EVSE cost, fuel cost, and GHG emission cost. EV owners have multiple strategies for charging their EVs, which can be classified as home, public places (e.g., workplaces, grocery stores, and shopping malls), or DC fast charging stations. Each charging location has corresponding capital and installation costs based on the available charging infrastructure and time-of-use electricity prices. Based on the levelized cost of charging model presented by Borlaug et al.,^10^ we expand the approach drawing on the levelized cost method to formulate the LCOO model by introducing the GHG emission cost, expressed as follows:

(Equation 1) $$LCOO_{k,i}^{\text{EV}}=\frac{c_{i}^{\text{capital}}+\sum_{y=1}^{L}\frac{c_{y,i}^{O\&M}}{{\left(1+dr\right)}^{y}}}{M\cdot L}+c_{k,i}^{\text{ele}}\cdot F_{k}^{\text{EV}}+c^{\text{carbon}}\cdot E_{k}^{\text{EV}}$$

(Equation 2) $$LCOO_{k}^{\text{EV}}=\sum_{i}w_{i}\cdot LCOO_{k,i}^{\text{EV}}$$

(Equation 3) $$LCOO_{k}^{\text{ICEV}}=c_{k}^{g}\cdot F^{\text{ICEV}}+c^{\text{carbon}}\cdot E^{\text{ICEV}}$$

(Equation 4) $$LCOO^{\text{EV}}=\frac{\sum_{k=1}^{K}LCOO_{1}^{\text{EV}}\cdot N_{1}^{\text{EV}}+...+LCOO_{k}^{\text{EV}}\cdot N_{k}^{\text{EV}}}{\sum_{k=1}^{K}N_{1}^{\text{EV}}+N_{2}^{\text{EV}}+...+N_{k}^{\text{EV}}}$$

where, $LCOO_{k,i}^{\text{EV}}$ represents the LCOO of EV at charging location i in province k, encompassing the EVSE cost, electricity cost, and GHG emission cost. The EVSE cost is dependent on the capital cost $c_{i}^{\text{capital}}$, the operating and maintenance cost $c_{y,i}^{O\&M}$, and the lifetime mileage of EV captured by the lifetime L and annual mileage M. dr represents the discount rate. The electricity cost is captured by the electricity price $c_{k,i}^{\text{ele}}$ and the fuel economy $F_{k}^{\text{EV}}$. The GHG emission cost is related to the environmental benefit, which is calculated by the carbon emission price $c^{\text{carbon}}$ (in $/ton) and GHG emission intensity $E_{k}^{\text{EV}}$ (in kg CO_2_/km). $w_{i}$ represents the weight coefficient at charging location i and the sum of $w_{i}$ is 1. $LCOO_{k}^{\text{EV}}$ represents the weighted sum of LCOO of EV at all charging locations in province k (in $/km). $LCOO_{k}^{\text{ICEV}}$ is the LCOO of gasoline ICEVs in province k (in $/km), including fuel cost and GHG emission cost. The fuel cost of ICEV is captured by the gasoline price $c_{k}^{g}$ and fuel economy $F^{\text{ICEV}}$. $E^{\text{ICEV}}$ is the well-to-wheel GHG emission intensity of gasoline ICEV (in kg CO_2_/km). $LCOO^{\text{EV}}$ represents the national average LCOO of EVs, which is a weighted average based on the private vehicle ownership of EV in each province. $N_{k}^{\text{EV}}$ represents the number of EVs in province k. K represents the number of provinces.

#### The fuel economy of EVs under different battery degradation conditions and temperatures

Fuel economy is notably affected by battery degradation and the ambient temperature. Battery degradation is characterized by decreases in energy density and electrochemical efficiency coupled with an increase in internal resistance.^56^ This process leads to an increase in energy consumption, ultimately limiting the mileage traveled by EV before recharging. In general, the ideal operational temperature range for a battery is 20°C to 30°C.^57^ However, the temperatures in various provinces of China exhibit considerable variations, with recorded extremes ranging from a high temperature of 40°C to a low temperature of -28°C. In instances with elevated ambient temperatures, there is an increased probability of the battery experiencing overheating, resulting in diminished efficacy in both the charging and discharging processes.^58^ The energy consumption of EV increases as the use of air conditioning to maintain occupant comfort levels in high-temperature and cold-weather areas increases. Low ambient temperatures increase motor and battery energy consumption while hindering regenerative energy recovery during driving.^57^^,^^58^ We use a linear function to show the relationship between the energy consumption of EV and different ambient temperatures according to publicly available data collected by the Canadian company FleetCarma^59^:

(Equation 5) $$\alpha_{T,k}=\left\{ \begin{array}{l} -0.0471\left(T+20.56\right)+1.46,\text{where}\;T\;<-20.56{}^{\circ}C\\ -0.0109T+1.24,\text{where}-20.56<T<0{}^{\circ}C\\ -0.0171\left(T-13.89\right)+1,\text{where}\;0<T<13.89{}^{\circ}C\\ 1,\text{where}\;13.89<T<23.89{}^{\circ}C\\ 0.0351\left(T-23.89\right)+1,\text{where}\;T\;>\;23.89{}^{\circ}C \end{array}\right.$$

(Equation 6) $$F_{k}^{\text{EV}}=\alpha_{T,k}\cdot \left(1-\eta \right)\cdot F^{s}$$

where $\alpha_{T,k}$ represents the energy consumption ratio at the monthly average temperature *T* in province k. $F^{s}$ represents the fuel economy under standard conditions (the standard value provided by the manufacturer), and η represents the annual battery degradation loss rate (assumed to be 0.71%^38^).

#### The GHG emissions of vehicles

GHG emission costs represent a substantial component of the LCOO, demonstrating the environmental benefits of driving EV. The well-to-wheel (WTW) GHG emissions associated with ICEV driving consist of both the emissions generated during fuel production (well-to-tank) and those emitted during vehicle usage (tank-to-wheel).^60^ The GHG emissions of EV include the GHG emissions produced by fossil fuel combustion in generators and upstream GHG emissions during fuel production.^37^ The GHG emissions that arise from fossil fuel combustion are significantly affected by the provincial generation mix.^61^ The consumption-based electricity mixes in each province based on interprovincial electricity trade is considered in the calculation of electricity emission intensity.^62^

The formula for the GHG emissions of ICEV (considering gasoline ICEV) is:

(Equation 7) $$E^{\text{ICEV}}=F^{\text{ICEV}}\left(EF^{W}+EF^{T}\right)$$

Where $E^{\text{ICEV}}$ represents the GHG emission intensity of gasoline ICEV (in kg CO_2_/km). $EF^{W}$ and $EF^{T}$ represent the well-to-tank factor of gasoline and the tank-to-wheel factor of gasoline (in kg CO_2_/L). $F^{\text{ICEV}}$ represents the fuel economy for gasoline (in km/L).

Based on the electricity emission intensity model proposed by Zhong Z et al.,^37^ the equations for the GHG emissions of EVs in each province are:

(Equation 8) $$E_{k}^{\text{EV}}=EF_{k}^{\text{ele}}\cdot F_{k}^{\text{EV}}$$

(Equation 9) $$EF_{k}^{\text{ele}}=GM_{k}\cdot RC\cdot \left(UE_{c}+BE_{c}\right)$$

where $E_{k}^{\text{EV}}$ represents the GHG emission intensity of EV in province k (in kg CO_2_/km). $EF_{k}^{\text{ele}}$ represents the GHG emissions factor of electricity in province k (in kg CO_2_/kWh), $F^{\text{EV}}$ is the fuel economy of EVs (in kWh/km). RC represents the coal consumption rate (in kg/kWh). $UE_{c}$ and $BE_{c}$ represent the upstream GHG emission factor and combustion emission factor (in kg CO_2_/kg standard coal). $GM_{k}$ represents the fossil energy generation share in province k.

### Quantification and statistical analysis

All statistical analyses of cost data were conducted using MATLAB 2022b. The visualization for simulation results was primarily created with MATLAB 2022b, as shown in Figures 1, 2, 4, 5, and 6. In addition, the visualization of the provincial levelized cost of EV operation was created with ArcMap 10.7, as shown in Figure 3.
