# Supplementary material for: A provincial cost analysis of electric vehicle operation in China
Source: iScience. 2025 Sep 7;28(10):113504. doi: 10.1016/j.isci.2025.113504 (PMC12538125; doi:10.1016/j.isci.2025.113504)
Supplement: Document S1. Figures S1–S6 and Tables S1–S6 [file mmc1.pdf]

iScience, Volume *28*

Supplemental information

A provincial cost analysis

# of electric vehicle operation in China

Bo Li, Mingxia Yang, Gang He, Guangchun Ruan, Jianxiao Wang, Xueqin Cui, Haiwang Zhong, and Daniel M. Kammen

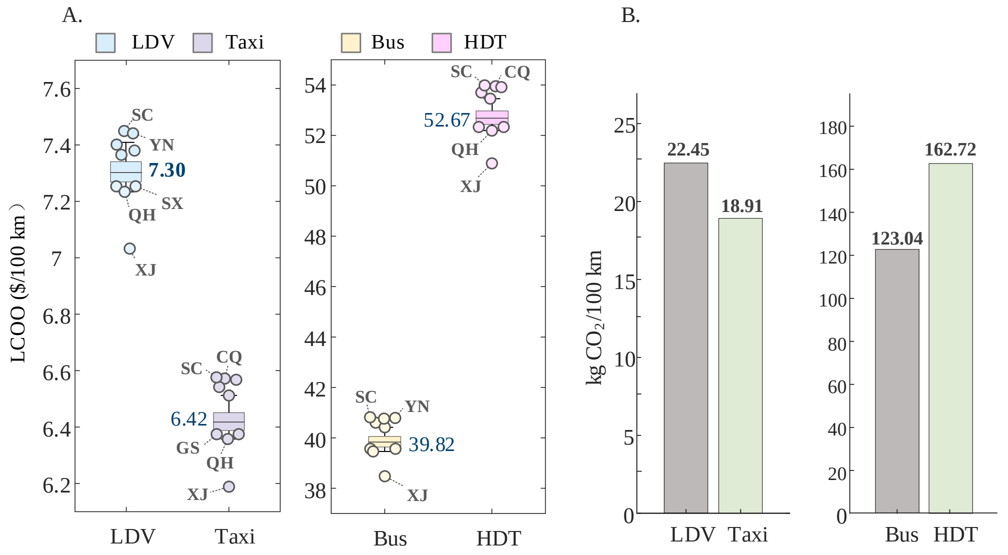

Figure S1. The provincial distribution of the LCOO and the GHG emission intensity for the four ICEV types in the baseline scenario. A: The boxplots show the distribution of the LCOO for the four types of ICEVs across provinces in China. Moreover, the provinces exhibiting significant deviations from the national average LCOO are highlighted with some data points. B: The GHG emission intensity for gasoline LDVs, taxis, buses, and HDTs in the baseline scenario.

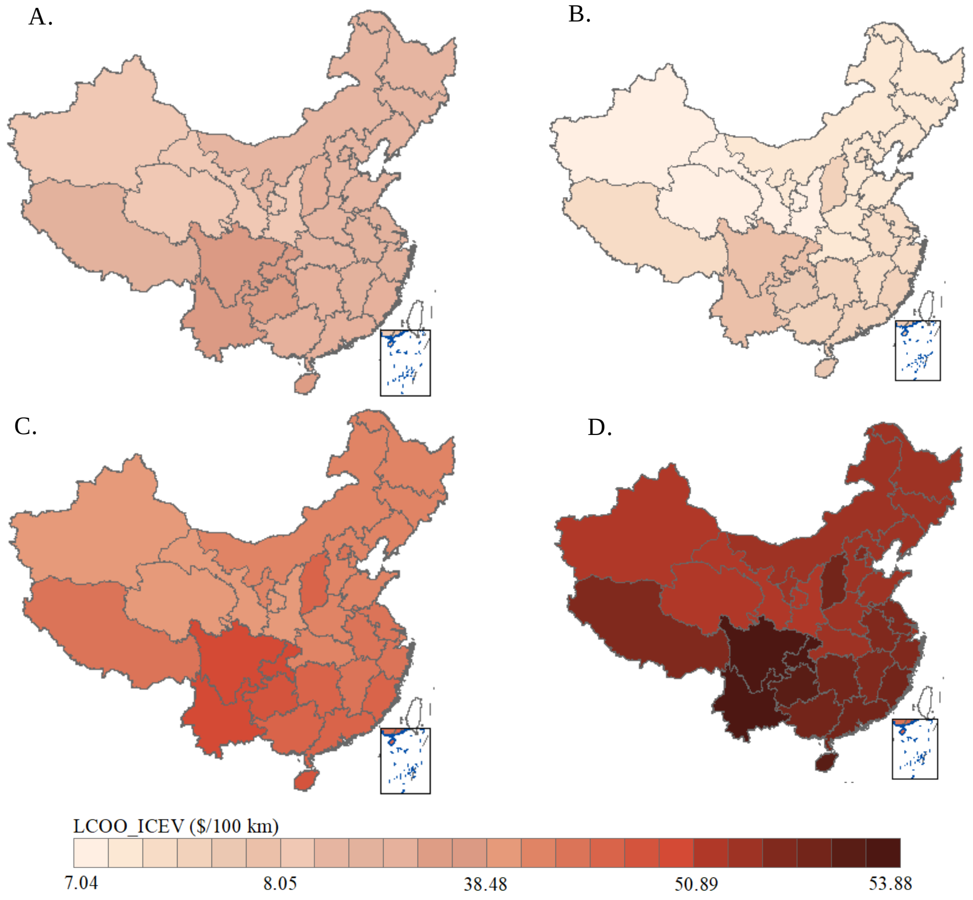

Figure S2. The provincial variation in the LCOO for the four ICEV types in the baseline scenario. A, LDVs. B, taxis. C, buses. D, HDTs.

Table S1. Vehicle tank-to-wheel fuel economies[1-4].

Vehicle Type	BEV (kWh/km)	GSL (L/km)	DSL (L/km)	VKT (km/year)
**Private LDVs**	0.134	0.0682	0.083	12000
**Public Buses**	1.14	0.372	0.315	120000
**Inter-city buses**	1.2	0.378	0.315	30000
**Taxis**	0.19	0.0600	0.083	120000
**Commercial LDPVs**	0.2	0.0986	0.1245	22000
**LDTs**	0.7	0.118	0.099	24000
**MDT**	1.1	0.204	0.17	32000
**HDTs**	1.46	0.492	0.34	40000

Note: GSL: gasoline; DSL: diesel; VKT: vehicle kilometer traveled; LDV: light-duty vehicle; LDT: light-duty truck; MDT: Medium-duty truck; HDT: heavy-duty truck.

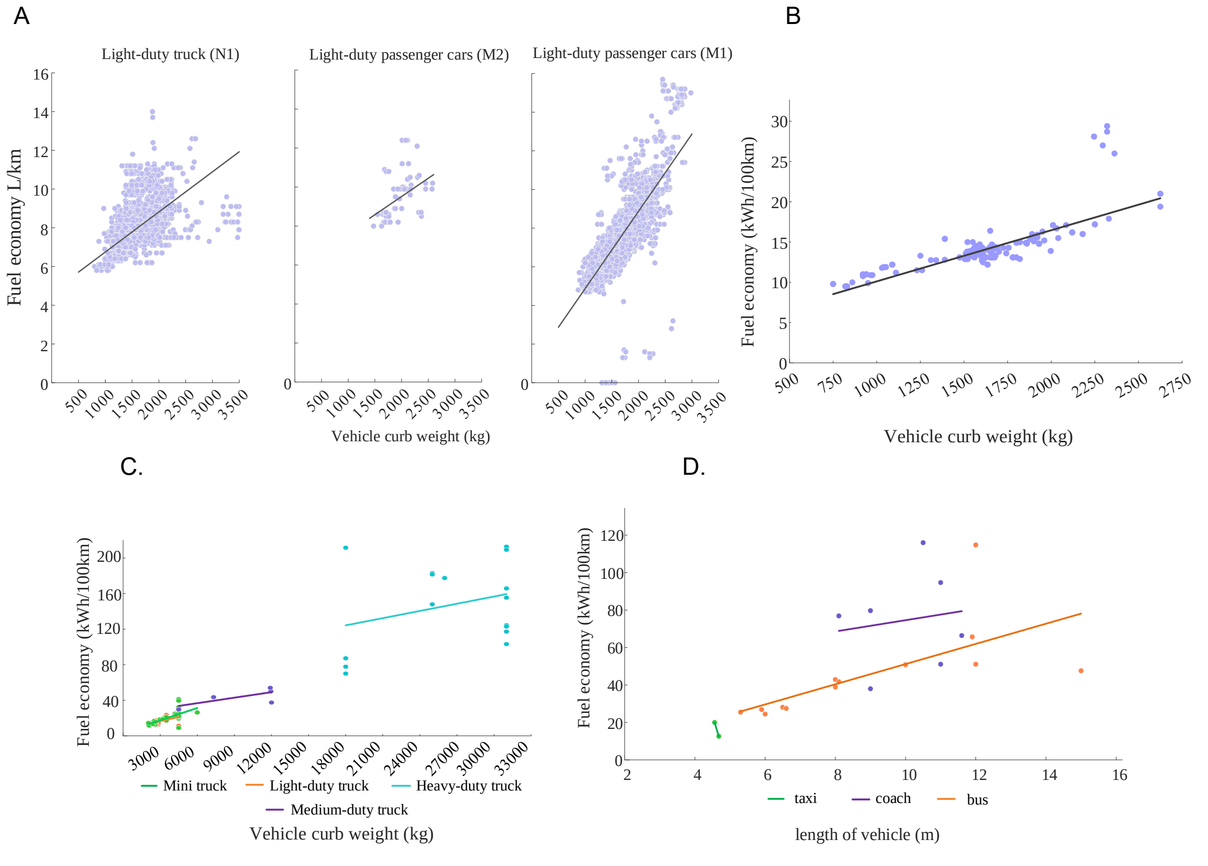

Figure S3. The FE for models of different vehicle types[5-8]. A. The Gasoline ICEVs from the Ministry of Industry and Information Technology of China between 2018 and 2021. B. The battery EVs from Auto Home. C. The battery electric trucks from Truck Home. D. The battery-electric buses from BYD, Yutong, and CRRC companies. The regression function for the ICEVs is shown as follows: FE(GVW) = 0.003998·GVW + 0.8734 for M1, FE(GVW)=0.001868·GVW + 5.782 for M2, FE(GVW)=0.002072· GVW + 4.676 for N1. The regression function for EVs from Auto Home is shown as follows: FE(GVW) = 0.0063465·GVW +3.775 for electric M1. The regression function for EVs from Truck Home is shown as follows: FE(GVW) = 0.003039·GVW +7.557 for electric mini-trucks, FE(GVW) = 0.004621·GVW +3.602 for electric light-truck, FE(GVW) = 0.002064·GVW +24.34 for electric medium truck, FE(GVW) = 0.002704·GVW +75.54 for electric heavy trucks).

Table S2. EVSE charging modes based on China standards GB/T 20234.1/2/3-2015[10,11,12].

Type	Voltage (V)	Charging Power (kW)	Items*	Places
L1	Single-phase AC 220	3.5	247	Residential
L2-1	Single-phase AC 220	7	572	Workplace, public parking place, et al.
L2-2	Three-phase AC 380	12	47	
L2-3	Three-phase AC 380	24	102	
L3-1	DC 600	60	30	Bus, taxi, and highway
L3-2	DC 600	120	27	

Table S3. Cost breakdown of EVSE from Shanghai and charging service companies[14].

EVSE type	Equipment cost ($/plug)	Installation cost ($/plug)	O&M costs ($/plug)
Public DC 60 kW	1957.71.43	29000	8371.49
Public DC 10 kW	4228.57	1714.29	1342.86
Public AC 3.5 kW	657.14	1069.71	614.29
Private AC 7 kW*	300 – 474.29	214.29 – 311.43	-

* The installation cost information comes from StarCharge[15] and TELD companies[16], which are the main charging service companies in China.

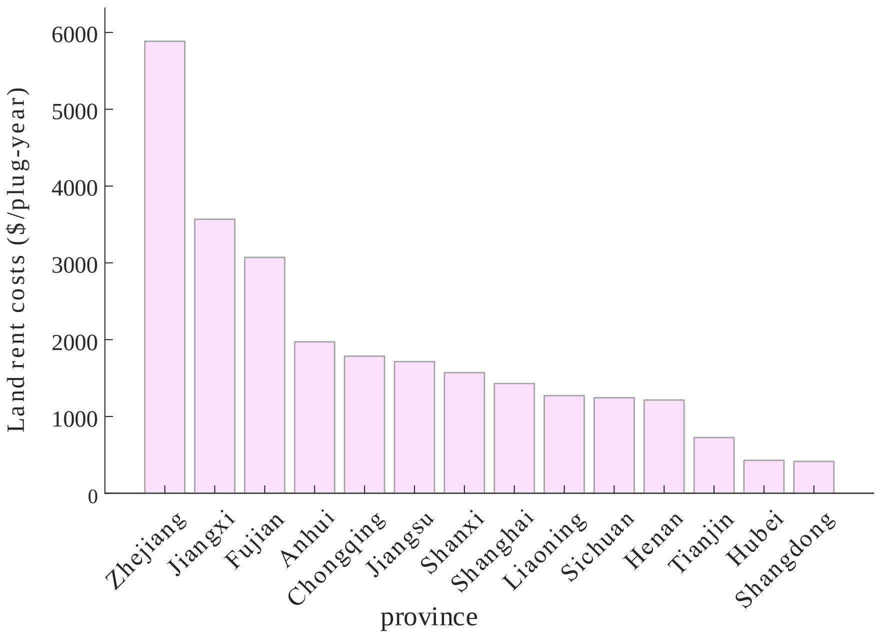

Figure S4. Provincial land rent costs of L3 EVSE projects along the highway.

Table S4. The planning targets of EVSE: EV ratio by EVSE type[17].

EVSE type	EVSE:EV ratio
Bus	1:1.6
Taxi	1:4
logistics	1:1
Private, dedicated, public institution	1:1

Table S5. The assumptions for EV chargers[18].

Mode	EVSE type	EVSE: EV ratio	Charging rate (kW)	Share of charging by EVSE type (%)
LDV	Private: L1	0.33	3.5	73%
	Public slow: L2	0.06	7	20%
	Public fast: L3	0.04	10	7%
Bus	Fast: L3	0.13*	60	50%
Truck	Fast: L3	0.26*	120	60%

* The EVSE:EV ratio of buses/trucks is the ratio of the number of EVSEs to the number of buses/trucks. LDVs represent private light-duty vehicles.

Table S6. Percent of total kWh for each charging location from the EPRI[19].

Location	Percent of total kWh
Home	81%
Work	6%
Public	3%
Other	10%

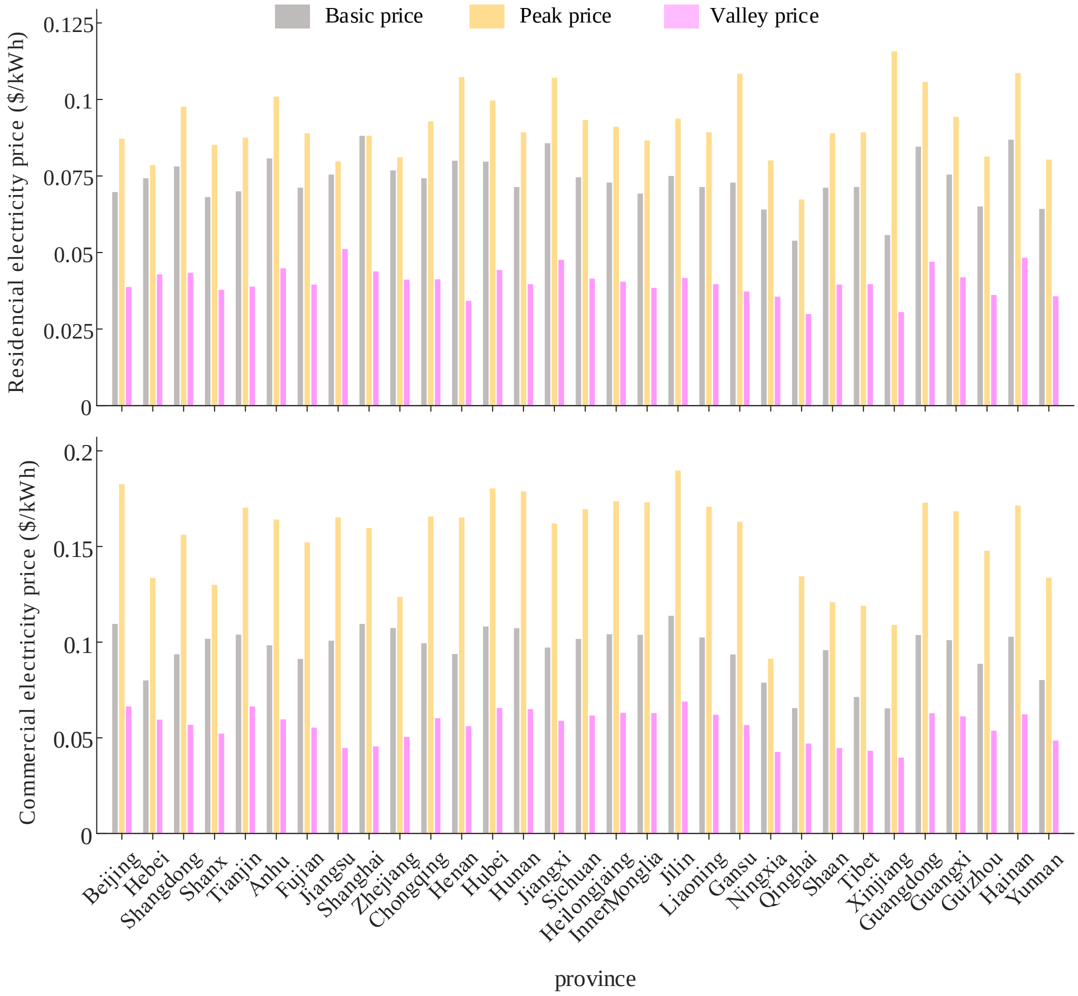

Figure S5.The electricity price by province in 2019[20].

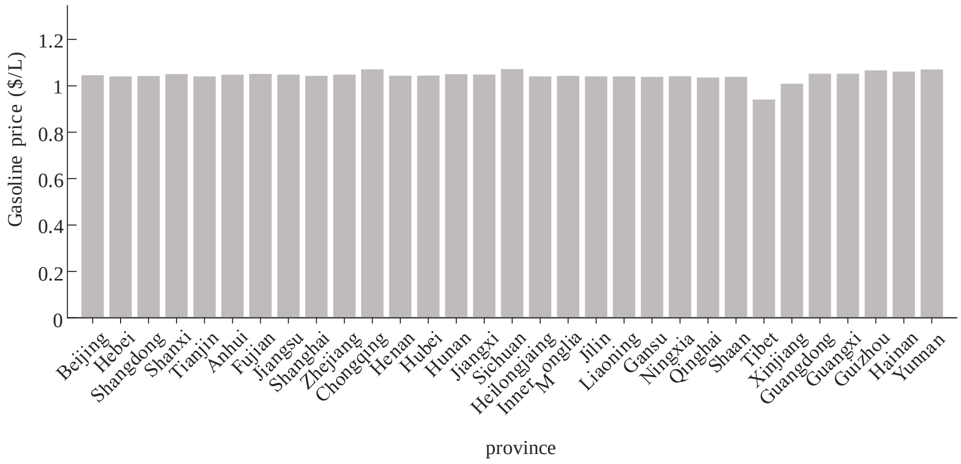

Figure S6.The gasoline prices derived from the NDRC[21].

Supplementary References

1. Ministry of Industry and Information Technology of the People's Republic of China (2021). Fuel consumption evaluation methods and targets for passenger cars. https://openstd. samr.gov.cn/bzgk/gb/newGbInfo?hcno=A0D5C7C6DE851F1FB293B6CA09C757EB
2. Ministry of Industry and Information Technology of the People's Republic of China (2020). Fuel consumption limits for passenger cars. https://openstd.samr.gov.cn/bzgk/gb/newGbInfo?hcno= 57DA347FDF48E4743786873E5B2D670A
3. Ministry of Industry and Information Technology of the People's Republic of China (2016). Limits of fuel consumption for light-duty commercial vehicles. http://www.gov.cn/xinwen/2016-01/20/content_5034692.htm.
4. Ministry of Industry and Information Technology of the People's Republic of China (2018). Fuel Consumption Limits for Heavy-Duty Commercial Vehicles. https://openstd.samr. gov.cn/bzgk/gb/newGbInfo?hcno=9C036161B1CEAFDA5225B7184A67229B.
5. Ministry of Industry and Information Technology of the People's Republic of China (2020). The average fuel consumption of passenger vehicles and the calculation of new energy vehicle credits in 2019. http://www.caam.org.cn/chn/1/cate_2/con_5230951.html.
6. Ministry of Industry and Information Technology of China (2021). China Automobile Fuel Consumption Inquiry System. https://yhgscx.miit.gov.cn/fuel-consumption-web/mainPage.
7. Autohome. https://www.autohome.com.cn/.
8. The Truck Home https://www.360che.com/.
9. Ministry of Industry and Information Technology of the People's Republic of China (2014). Classification of power-driven vehicles and trailers.
10. Ministry of Industry and Information Technology of the People's Republic of China (2023). Connection set for conductive charging of electric vehicles—Part 1: General requirements. https://openstd.samr.gov.cn/bzgk/gb/newGbInfo?hcno=5A456919C33E2C9041B843AB3D36AB6 7.
11. Ministry of Industry and Information Technology of the People's Republic of China (2023). Connection set for conductive charging of electric vehicles—Part 3: DC charging couple. https://openstd.samr.gov.cn/bzgk/gb/newGbInfo?hcno=5928F89DE3DB6FD3FDDC06E709FCC4 A2
12. Ministry of Industry and Information Technology of the People's Republic of China (2015). Connection set for conductive charging of electric vehicles—Part 2: AC charging coupler. https://openstd.samr.gov.cn/bzgk/gb/newGbInfo?hcno=1CABFC2874B7A9C7089442BF8A90CD DA
13. JD online shopping website https://www.jd.com/.
14. Pu J. (2019). Operation Cost Accounting and Economic Benefit Analysis of Bus Charging Stations and Public Charging Piles. Shanghai Energy Conservation, 70–76.
15. StarCharge. company https://www.starcharge.com/en/home.
16. TELD. company https://www.teld.cn/HomePage/Index.
17. National Development and Reform Commission (2015). Guidelines for the development of electric vehicles charging infrastructure (2015-2020). https://www.gov.cn/zhengce/2015-10/09/content_ 5076250.htm.
18. IEA (2022). Global EV Outlook 2022. https://www.iea.org/reports/global-ev-outlook-2022.
19. Electric Power Research Institute (2018). Electric Vehicle Driving, Charging, and Load Shape

Analysis: A Deep Dive Into Where, When, and How Much Salt River Project (SRP) Electric Vehicle Customers Charge (Electric Power Research Institute (EPRI)).
20. National Development and Reform Commission https://www.ndrc.gov.cn/.
21. National Development and Reform Commission (2020). The highest retail prices of gasoline and diesel in various provinces. https://www.ndrc.gov.cn/xwdt/ztzl/gncpyjg/202012/t20201217_1293038.html.
